# Advancing bladder cancer management: development of a prognostic model and personalized therapy

**DOI:** 10.3389/fimmu.2024.1430792

**Published:** 2024-07-22

**Authors:** Xiang Huang, Guotu Du, Ying Yang, Peng Su, Shicheng Chen, Chongjiong Cai, Tianyu Huang, Yu Zeng, Yonggang Tao, Demei Tian, Neng Zhang

**Affiliations:** ^1^ Department of Urology, Affiliated Hospital of Zunyi Medical University, Zunyi, China; ^2^ Department of Pathology, Affiliated Hospital of Zunyi Medical University, Zunyi, China; ^3^ Department of Urology, The Second Affiliated Hospital of Zunyi Medical University, Zunyi, China; ^4^ Department of Urology, Renhuai People’s Hospital, Zunyi, China; ^5^ Department of Nursing, Affiliated Hospital of Zunyi Medical University, Zunyi, China

**Keywords:** bladder cancer, molecular subtypes, basal squamous, prognostic model, single-cell RNA sequencing, personalized treatment

## Abstract

**Background:**

Bladder cancer (BLCA) was recognized as a significant public health challenge due to its high incidence and mortality rates. The influence of molecular subtypes on treatment outcomes was well-acknowledged, necessitating further exploration of their characterization and application. This study was aimed at enhancing the understanding of BLCA by mapping its molecular heterogeneity and developing a robust prognostic model using single-cell and bulk RNA sequencing data. Additionally, immunological characteristics and personalized treatment strategies were investigated through the risk score.

**Methods:**

Single-cell RNA sequencing (scRNA-seq) data from GSE135337 and bulk RNA-seq data from several sources, including GSE13507, GSE31684, GSE32894, GSE69795, and TCGA-BLCA, were utilized. Molecular subtypes, particularly the basal-squamous (Ba/Sq) subtype associated with poor prognosis, were identified. A prognostic model was constructed using LASSO and Cox regression analyses focused on genes linked with the Ba/Sq subtype. this model was validated across internal and external datasets to ensure predictive accuracy. High- and low-risk groups based on the risk score derived from TCGA-BLCA data were analyzed to examine their immune-related molecular profiles and treatment responses.

**Results:**

Six molecular subtypes were identified, with the Ba/Sq subtype being consistently associated with poor prognosis. The prognostic model, based on basal-squamous subtype-related genes (BSSRGs), was shown to have strong predictive performance across diverse clinical settings with AUC values at 1, 3, and 5 years indicating robust predictability in training, testing, and entire datasets. Analysis of the different risk groups revealed distinct immune infiltration and microenvironments. Generally higher tumor mutation burden (TMB) scores and lower tumor immune dysfunction and exclusion (TIDE) scores were exhibited by the low-risk group, suggesting varied potentials for systemic drug response between the groups. Finally, significant differences in potential systemic drug response rates were also observed between risk groups.

**Conclusions:**

The study introduced and validated a new prognostic model for BLCA based on BSSRGs, which was proven effective in prognosis prediction. The potential for personalized therapy, optimized by patient stratification and immune profiling, was highlighted by our risk score, aiming to improve treatment efficacy. This approach was promised to offer significant advancements in managing BLCA, tailoring treatments based on detailed molecular and immunological insights.

## 1 Introduction

Within the spectrum of genitourinary malignancies, bladder cancer (BLCA) stands out as one of the most frequently encountered, with a noted predilection for males over females. Most patients are diagnosed between the ages of 50 and 70 (1, 2). Globally, BLCA ranks fourth in newly diagnosed male cancer cases and has risen to the eighth position in terms of mortality (3). BLCA exhibits heterogeneous clinical behaviors, stratified by the extent of tumor invasion into bladder tissues, which broadly delineates into non-muscle invasive bladder cancer (NMIBC) and the more advanced muscle-invasive bladder cancer (MIBC) (4). Within it, MIBC, constituting about 25% of cases, is characterized by high malignancy, rapid progression, early metastasis, and a high recurrence and mortality rate (5). Currently, for MIBC, radical surgery following neoadjuvant therapy is recommended. However, the efficacy of this treatment regimen has not reached an ideal level (6). Therefore, early prediction of patient survival prognosis and personalized adjuvant therapy, which are based on different tumor characteristics, have important practical significance.

Recent studies have indicated significant heterogeneity in the molecular mechanisms underlying the occurrence and development of MIBC (7). Tumors dominated by different subtypes may lead to different treatment outcomes and prognoses, which could be one of the reasons for the high heterogeneity of BLCA (8). Many classifications of bladder molecular subtypes exist, however, further studies and applications of these subtypes are comparatively sparse (9–12). Nevertheless, distinct differentiation patterns, carcinogenic mechanisms, tumor microenvironments, and significant histological and clinical relevance are exhibited by molecular subtypes, which are crucial for addressing the heterogeneity of BLCA, predicting treatment efficacy, and determining prognosis. The application of tumor subtypes is worth further exploration.

The advanced use of single-cell RNA sequencing (scRNA-seq) technology enables us to explore the heterogeneity of different subtypes of bladder tumors at the individual cell level based on transcriptomic changes and to establish prognostic prediction models (13), Moreover, bulk RNA can confirm the results of single-cell analyses at the overall genetic level, verifying the effectiveness of the predictive models in larger samples, as well as to investigate the responses of subtypes to treatment (14, 15). Therefore, by integrating both approaches, we can not only develop tools for predicting BLCA prognosis based on molecular subtypes, but also explore drug sensitivity testing based on molecular subtypes, providing support for personalized treatment.

Recent decades have seen significant advancements in a variety of systemic cancer therapies. The emergence of immune checkpoint inhibitors (ICIs) has brought about significant changes, becoming a secondary treatment option for those who lose the chance of radical resection and for patients with metastatic BLCA, especially in cases where platinum-based chemotherapy is not suitable for first-line treatment (16). However, A response rate of around 20% is typically achieved by ICIs in the treatment of MIBC (17). Whether efficacy is affected by tumor subtypes is worth further investigation.

This study integrates scRNA-seq data and bulk RNA-seq data to comprehensively analyze BLCA, initially identifying the subtypes of BLCA. Subsequently, a prognostic model based on basal-squamous subtype-related genes (BSSRGs) was constructed and verified through internal and external validation cohorts. Lastly, molecular and immune analyses reveal insights into BLCA biology, highlighting potential therapeutic targets and supporting personalized treatments.

## 2 Materials and methods

### 2.1 Preparation for RNA-seq data

**Figure 1:**
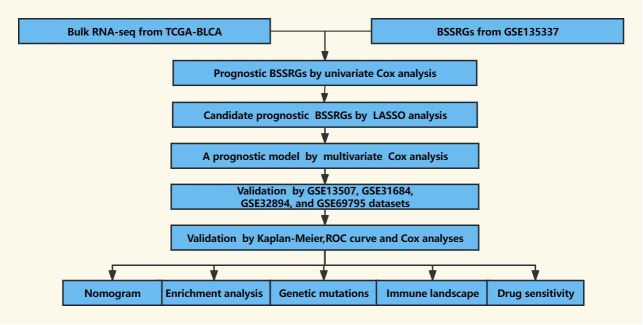
The workflow of the present study.

The scRNA-seq data for BLCA was obtained from the GSE135337 (Gene Expression Omnibus dataset), which includes seven BLCA tissue samples and one paracancerous tissue sample (18). To investigate gene expression signatures and potential prognostic factors in BLCA comprehensively, bulk RNA-seq data and accompanying clinical information were collected from multiple datasets, including The Cancer Genome Atlas-Bladder Urothelial Carcinoma (TCGA-BLCA), GSE13507, GSE31684, GSE32894, and GSE69795 (12, 19–23). Only samples with survival times exceeding 30 days were retained to ensure the reliability of the analyses, allowing for sufficient follow-up and observation of the clinical course of the disease. A comprehensive dataset was compiled, comprising 7 BLCA samples from GSE135337, 391 BLCA samples from TCGA-BLCA, 165 BLCA samples from GSE13507, 93 BLCA samples from GSE31684, 224 BLCA samples from GSE32894, and 38 BLCA samples from GSE69795. A schematic outline of the study workflow is depicted in **Figure 1**.

### 2.2 Processing of scRNA-seq data

The scRNA-seq data was analyzed using the R package “Seurat” (v4.3.0) (24). The scRNA-seq data was analyzed using the R package “Seurat” (v4.3.0) (24). The quality control of scRNA-seq data was conducted following the methodology described in a previous study (18). Subsequently, cell cycle effects were removed, normalization and dimensionality reduction (1:40) were applied, and clustering (resolution=1) and cell annotation were performed on the Seurat objects (25). Specifically, six distinct malignant epithelial cell subtypes—namely basal-squamous (Ba/Sq), luminal, stress, metabolism, cell cycle, and immunity—were identified using the “FindAllMarkers” function, which allowed for the identification of highly variable genes in each subtype. The various differentiation states of the malignant epithelial cell subpopulations were analyzed using “Monocle 2” (26). The degree of transcription factor activation across these subtypes was evaluated using “pySCENIC” (27). Finally, communication patterns were examined using the “CellChat” tool, which leveraged ligand-receptor information to model communication probability. Through this analysis, significant communication events were successfully identified (28).

### 2.3 Development and validation of prognostic model

A survival analysis was conducted to investigate the impact of different malignant epithelial cell subtypes on the prognosis of BLCA. Genes that displayed high variability specifically within the basal-squamous subtype were identified and designated as BSSRGs. Initially, a univariate Cox analysis was conducted to assess the prognostic significance of these BSSRGs, with a significance threshold set at P<0.05. The BLCA samples from the TCGA-BLCA dataset were randomly divided into a training set and an internal validation set in a 7:3 ratio for our study cohort. Additionally, BLCA samples from external datasets, namely GSE13507, GSE31684, GSE32894, and GSE69795, were utilized as an external validation set to further validate the findings. Next, LASSO analysis was employed to identify candidate BSSRGs. Subsequently, a prognostic model based on these candidate genes was established through multivariate Cox analysis. To evaluate the accuracy of this model in predicting prognosis, a risk score for each patient was calculated using the specified formula: $\sum_{\text{i}=1}^{\text{k}}\text{β}\text{i}\text{S}i$ . 

Kaplan-Meier analysis and the chi-squared test were utilized to assess the prognostic model’s capacity to distinguish survival disparities amongst various risk score groups. The time-dependent receiver operating characteristic (ROC) curve was utilized to estimate the model’s predictive ability compared to typical clinical characteristics. To verify the applicability of this model to patients with disparate clinical features, survival dissimilarities between varied risk score groups within every subgroup were compared. Univariate and multivariate Cox analyses were performed to assess the model as an independent indicator of prognosis. The consistency index (C-index) was utilized to estimate the predictive power of the signature in comparison with clinical features. A nomogram incorporating the prognostic model and clinical features was created to predict one, three, and five-year survival rates of BLCA patients.

### 2.4 Enrichment analysis and gene mutation analysis

The molecular mechanisms and pathways associated with different risk score groups were explored by identifying differentially expressed genes (DEGs) between these groups, using criteria of |logFC≥1| and FDR<0.05. Gene Ontology (GO) and Kyoto Encyclopedia of Genes and Genomes (KEGG) analyses were performed to further investigate these associations (P<0.05) (29, 30). Additionally, the frequency of mutations in different risk score groups was quantified using the R package “Maftools” (31).

### 2.5 Assessment of tumor immune microenvironment

A range of algorithms including TIMER, XCELL, QUANTISEQ, MCP-COUNTER, EPIC, CIBERSORT-ABS, and CIBERSORT were utilized to evaluate immune cell infiltration (32–38). Differences in immune function were examined by conducting a single-sample Gene Set Enrichment Analysis (ssGSEA) (39). Additionally, the expression levels of various immune checkpoint genes were investigated. Mutational analysis was carried out to determine the number of gene mutations. Tumor immune dysfunction and exclusion (TIDE) scores and tumor mutation burden (TMB) scores were calculated to predict the response to immunotherapy (40, 41).

### 2.6 Identification of anti-tumor medicines

The potential effectiveness of various drugs in treating BLCA was assessed using the “oncoPredict” tool to predict drug responsiveness (42). Promising medications for BLCA treatment were successfully identified through this analysis by evaluating the Genomics of Drug Sensitivity in Cancer (GDSC) database.

### 2.7 Western blot and immunohistochemistry

Tissue samples were homogenized using RIPA buffer (Solarbio, Beijing, China) supplemented with protease and phosphatase inhibitors. Protein concentration was determined using a BCA protein assay kit (Solarbio, Beijing, China). Equal amounts of protein samples were loaded onto SDS-PAGE gels and separated by electrophoresis. Proteins were transferred onto PVDF membranes using a wet transfer system. Membranes were blocked in blocking buffer (EpiZyme, Shanghai, China) for 1 hour at room temperature. Membranes were incubated with SUMF2(1:4000, Proteintech, Wuhan, China), KDELR2 (1:2000, HUABIO, Hangzhou, China), TM4SF1 (1:1000, Youpin Biotech, Shenzhen, china), and SCD (1:1000, ZENBIO, Chengdu, China) overnight at 4°C. After washing, membranes were incubated with HRP-conjugated secondary antibodies for 1 hour at room temperature. Protein bands were visualized using the Super ECL Detection system and imaged with a chemiluminescence detection system (Biosharp, Anhui, China).

Paraffin-embedded tissue sections (4 μm thick) were deparaffinized in xylene and rehydrated through a graded alcohol series. Antigen retrieval was performed using citrate buffer (pH 6.0) in a pressure cooker. Endogenous peroxidase activity was blocked with 3% hydrogen peroxide for 10 minutes, and non-specific binding was blocked with 5% normal goat serum for 1 hour. Sections were incubated with SUMF2(1:200, Proteintech, Wuhan, China), KDELR2 (1:200, HUABIO, Hangzhou, China), TM4SF1 (1:200, Youpin Biotech, Shenzhen, china), and SCD (1:100, ZENBIO, Chengdu, China) overnight at 4°C. After washing, sections were incubated with biotinylated secondary antibodies for 30 minutes at room temperature. Signal was developed using the DAB substrate kit (elabscience, Wuhan, China), and sections were counterstained with hematoxylin. Sections were dehydrated, cleared, and mounted with coverslips.

### 2.8 Statistical analysis

Data analysis and the creation of figures were performed using R software, version 4.2.1, available at www.R-project.org. Statistical significance was defined as p-value< 0.05.

## 3 Result

### 3.1 Comprehensive single-cell analysis of BLCA samples

**Figure 2:**
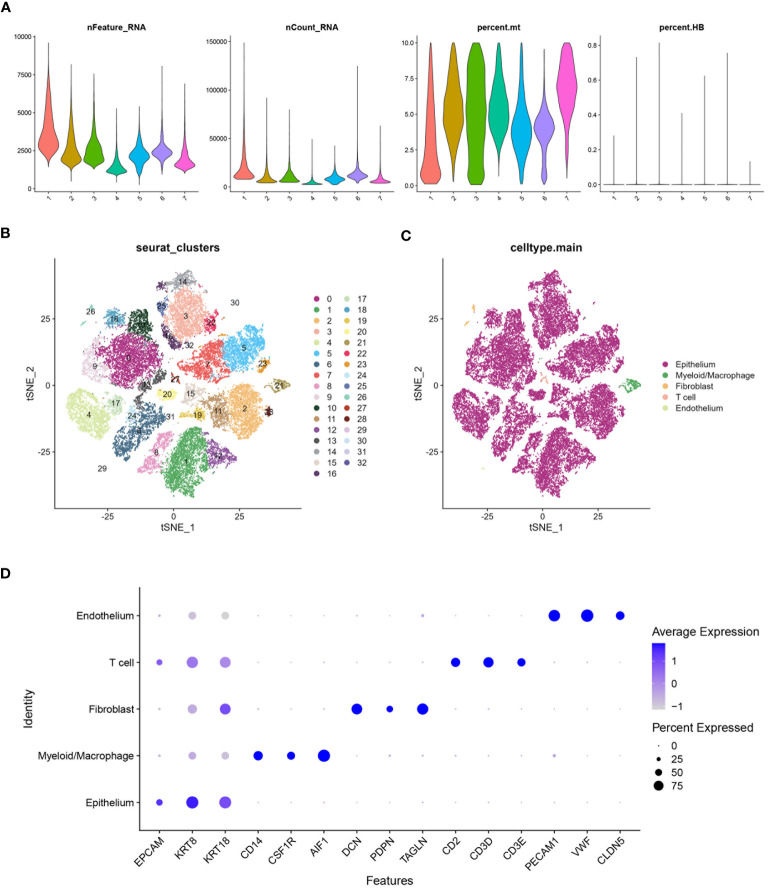
Processing of scRNA data. **(A)** Violin plot shows the distribution of several parameters across seven BLCA samples from the scRNA database GSE135337. **(B)** tSNE plot shows all the single cells in seven BLCA samples can be classified into thirty-two clusters. **(C)** tSNE plot categorizing the thirty-two clusters into five main cell types: Epithelium, Myeloid/Macrophage, Fibroblast, T cell, and Endothelium. **(D)** Bubble chart highlighting the characteristic genes associated with different cell types. The size of the bubbles represents the percentage of cells expressing the gene, and the color intensity indicates the average expression level.

**Figure 3:**
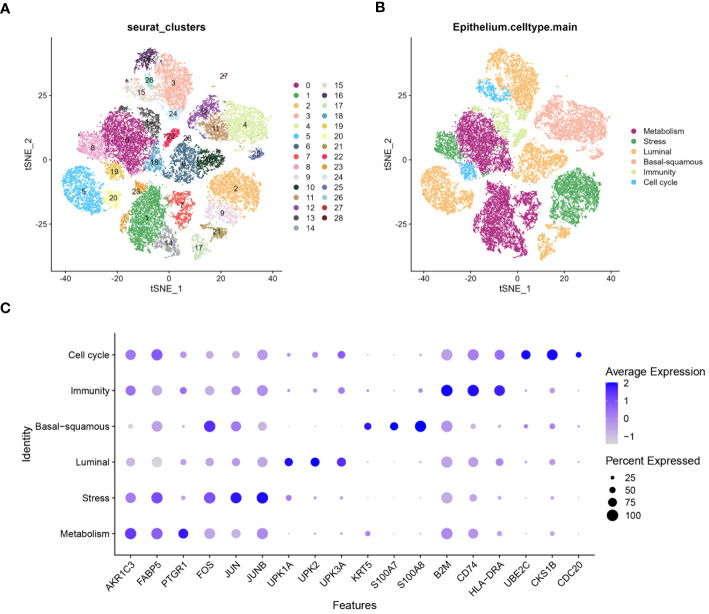
Characterization of epithelium diversity and molecular subtypes. **(A)** tSNE plot suggests epithelial cell can be classified into twenty-eight clusters. **(B)** tSNE plot identifying six molecular subtypes of epithelial cells from the twenty-eight clusters: metabolism, stress, luminal, basal-squamous, immunity, and cell cycle. **(C)** Bubble chart highlighting the highly variable genes associated with different molecular subtypes of epithelial cells. The size of the bubbles represents the percentage of cells expressing the gene, while the color intensity indicates the average expression level.

A comprehensive analysis of BLCA samples at the single-cell level was conducted, with data being extracted from a scRNA-seq dataset (GSE135337). Standards for quality control were established to ensure data reliability (standards: minGene=200, maxGene=10000, minUMI=500, pctMT=10, pctHB=1). nFeature_RNA showed the number of detected genes among samples. nCount_RNA displayed the total RNA count per cell, highlighting differences in cellular RNA content. Percent.mt represented the percentage of mitochondrial gene expression, used as a quality control metric to assess cell viability. Percent.HB indicated the percentage of hemoglobin gene expression, helping to identify potential contamination by red blood cells (**Figure 2A**). Thirty-two clusters were identified through the analysis of seven BLCA samples (**Figure 2B**). These clusters were further classified into five cell types: epithelium, myeloid/macrophage, fibroblast, T cell, and endothelium, with epithelial cells forming the majority and dominating the landscape (**Figure 2C**). The expression of characteristic genes associated with each cell type were identified, such as KRT8, KRT18, and EPCAM are predominantly expressed in epithelial cells (**Figure 2D**). Further analysis focused on the epithelial cells within the BLCA samples, revealing significant diversity and identifying key molecular subtypes. The extraction and clustering of malignant epithelial cells were carried out, revealing twenty-eight clusters (**Figure 3A**). These clusters were further classified based on gene expression patterns, which enabled the identification of six distinct cell types: metabolism, stress, luminal, basal-squamous, immunity, and cell cycle (**Figure 3B**). The top variable genes for each cell subtype were highlighted in the plot, with FOS, S100A8, S100A7, and KRT5 being the most expressed in the Ba/Sq subtype (**Figure 3C**).

### 3.2 Dynamics of epithelial cell states and intercellular communication

**Figure 4:**
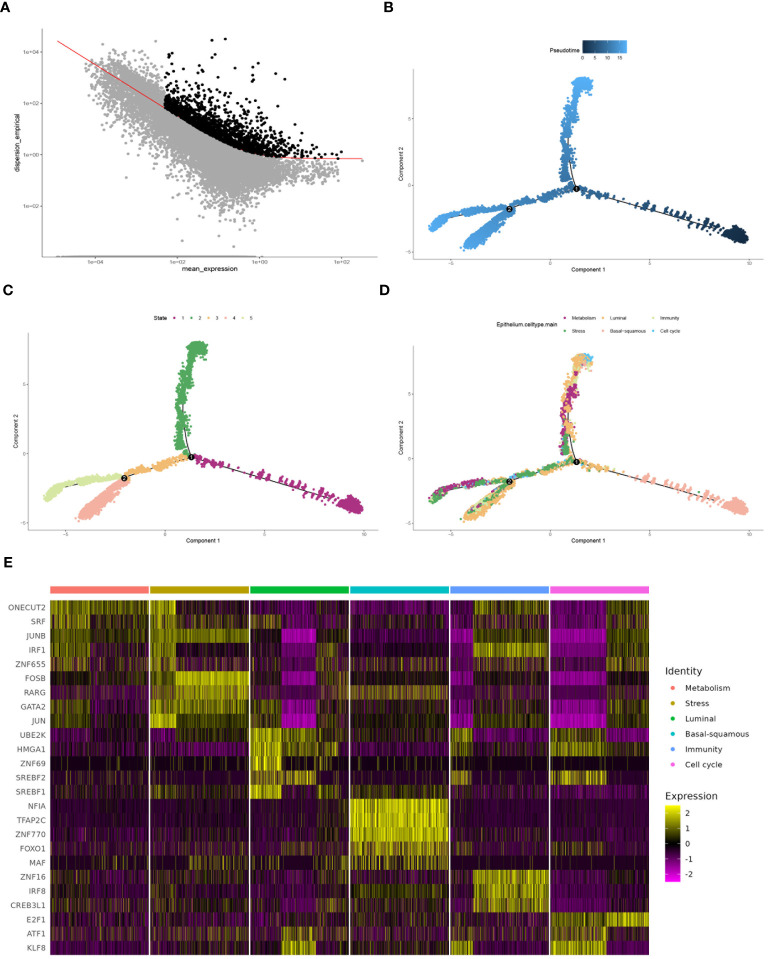
Comprehensive analysis of cell differentiation dynamics. **(A)** Selecting high dispersion genes for trajectory construction by monocle. **(B)** Pseudotime analysis depicting different stages of cell differentiation. **(C)** Plot showing the state of cells during the differentiation process, analyzed by pseudotime trajectory analysis. **(D)** Plot illustrating the progression of each epithelial cell subtype along the differentiation trajectory. **(E)** Heatmap demonstrating transcription factor activation across different molecular subtypes, with the color intensity indicating the level of expression.

**Figure 5:**
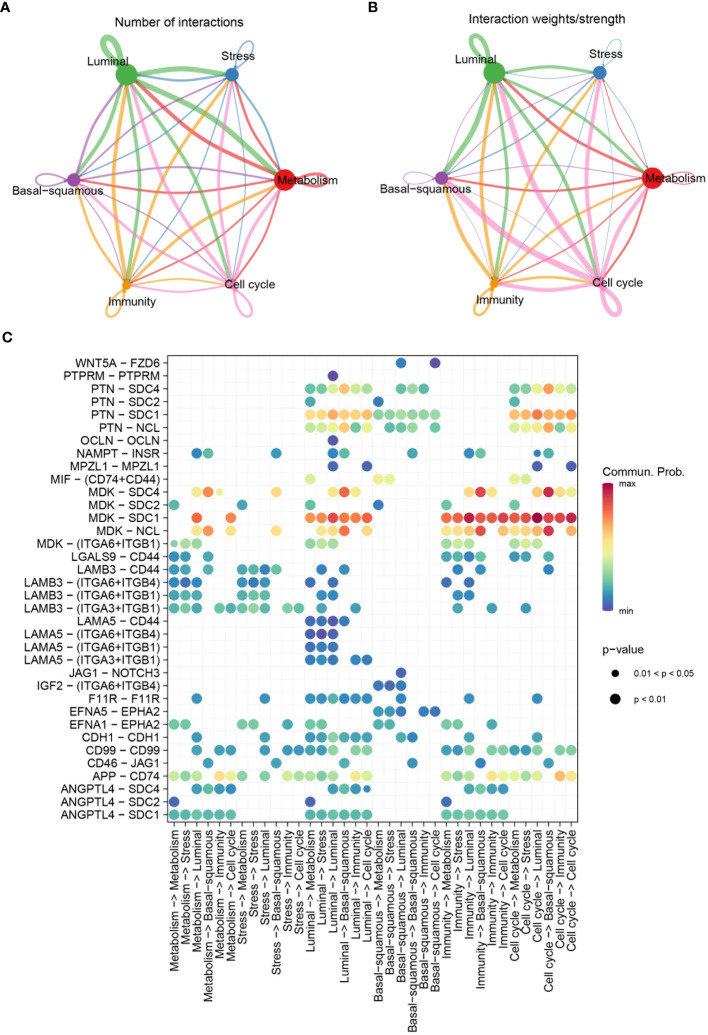
Communication patterns among molecular subtypes. **(A)** Diagram depicting the number of interactions among different molecular subtypes. **(B)** Diagram showing the interaction weights/strengths among the subtypes. **(C)** Bubble chart visualizing significant communicated ligand-receptor pairs among molecular subtypes. The size of the bubbles represents the p-value, with smaller bubbles indicating more significant interactions, and the color intensity represents the communication probability, with higher values indicating stronger communication.

Highly variable genes were systematically identified from the gene expression profile of epithelial cells to construct a trajectory (**Figure 4A**). Subsequently, pseudotime trajectory illustrated the progression of cells along a differentiation timeline, with cells transitioning from early to late differentiation stages (**Figure 4B**). The epithelial cells were effectively stratified into five distinct states through cells progress during differentiation (**Figure 4C**). Concurrently, the differential distribution of each epithelial cell subtype in pseudotime analysis was clearly depicted (**Figure 4D**). Furthermore, the activation patterns of transcription factors within each subtype of epithelial cells have been thoroughly investigated. Expression of genes such as FOSB, GATA2, and JUN had been observed to increase in the stress subtype, while a noticeable decrease had been observed in the luminal and cell cycle subtypes (**Figure 4E**). An intercellular communication network among different subtypes of epithelial cells was established, quantifying the number of interactions, the metabolism subtype demonstrated a high number of interactions with other subtypes, indicating its central role in cellular communication (**Figure 5A**). Stronger interactions were observed between the metabolism subtype and other subtypes, such as cell cycle and stress subtypes (**Figure 5B**). Additionally, the communication between various molecular subtypes was primarily mediated through ligand-receptor interactions involving MDK-SDC1, MDK-NCL, and MDK-SDC4. Communication from other subtypes to Ba/Sq was predominantly conveyed through these three interactions. In contrast, communication from Ba/Sq to other subtypes was characterized by a relatively weak probability, primarily involving PTN-SDC1. This detailed mapping of ligand-receptor interactions underscores the complex communication network within BLCA, providing valuable insights into potential therapeutic targets and strategies for disrupting tumor-promoting signals (**Figure 5C**).

### 3.3 Prognostic model development for BLCA

**Figure 6:**
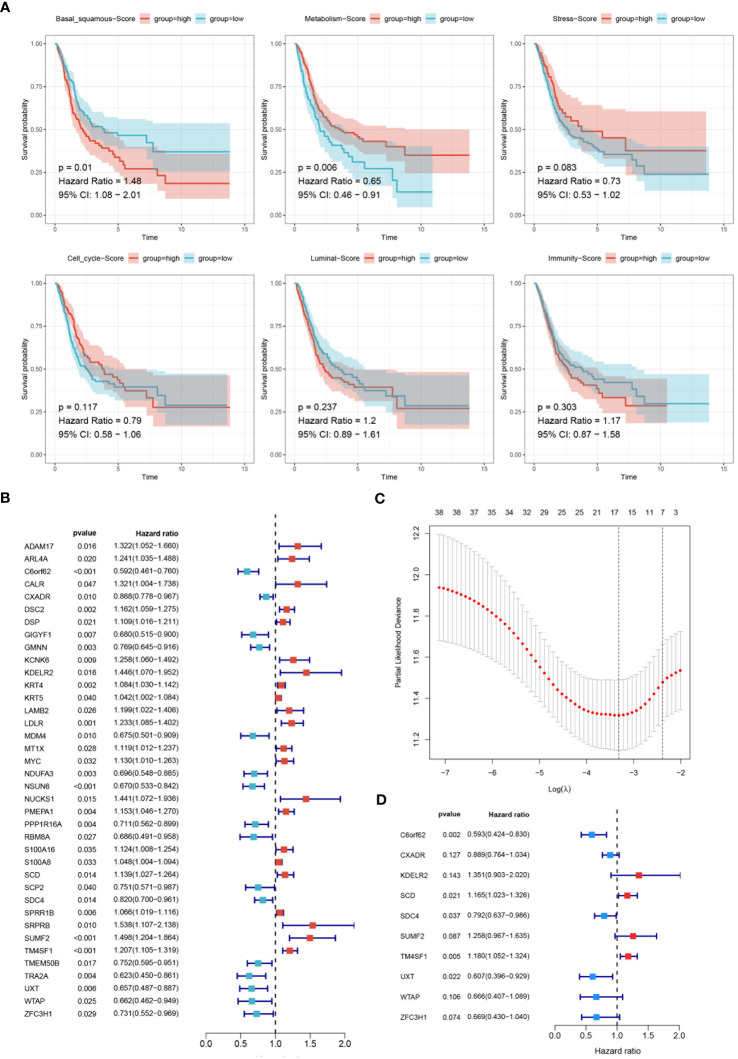
Development of the prognostic model. **(A)** Survival analysis evaluating the impact of six molecular subtypes (Basal-squamous, Metabolism, Stress, Cell cycle, Luminal, and Immunity) on the prognosis of BLCA, depicted through Kaplan-Meier survival curves. The p-values and hazard ratios with 95% confidence intervals are indicated. **(B)** Forest plot displaying the results of univariate Cox analysis to evaluate BSSRGs with prognostic significance. **(C)** LASSO analysis illustrating the trajectories and distributions for each independent variable regarding lambda. **(D)** A forest plot generated from multivariate Cox analysis was used to identify BSSRGs.

Kaplan-Meier analysis was utilized to evaluate the impact of six molecular subtypes on the prognosis of BLCA. A significant statistical difference was observed between the basal-squamous score group and the metabolism score group, while stress, cell cycle, luminal and immunity scores showed no significant difference. Patients in the low score group of basal-squamous (HR=1.48, P=0.01) and the high score group of metabolism (HR=0.65, P=0.006) showed better survival outcomes (**Figure 6A**). Subsequently, univariate Cox analysis identified thirty-eight genes significantly associated with prognosis, with twenty-two genes HR>1 (**Figure 6B**). LASSO analysis illustrated the trajectories and distributions for each independent variable with respect to lambda. The optimal lambda value was identified to balance model complexity and predictive performance (**Figure 6C**). BSSRGs were identified by multivariate Cox analysis, including C6orf62, CXADR, KDELR2, SCD, SDC4, SUMF2, TM4SF1, UXT, WTAP, ZFC3H1 (**Figures 6D**).

**Figure 7:**
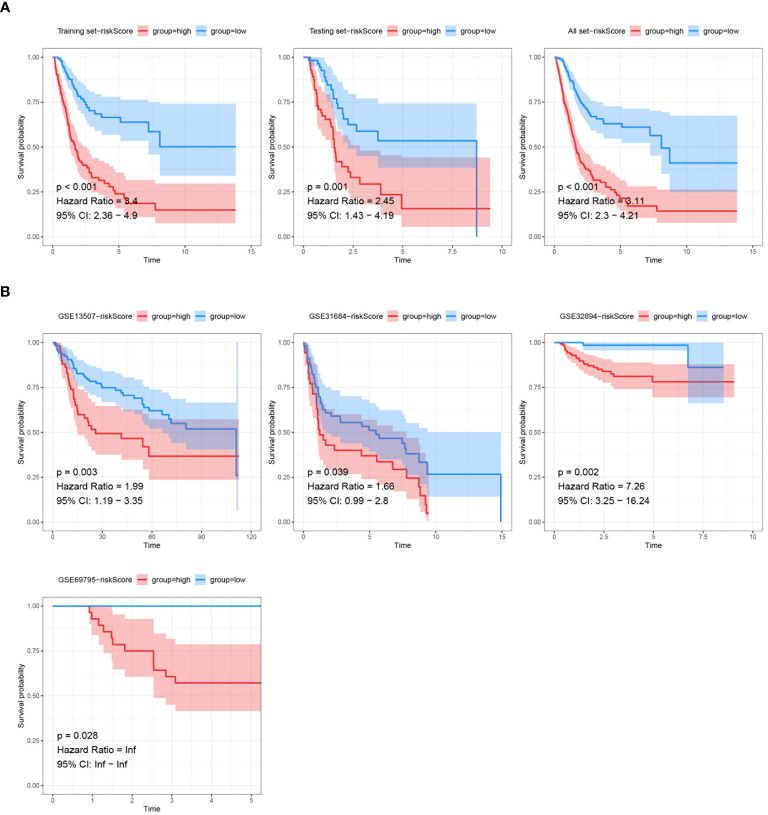
Validation of prognostic models via Kaplan-Meier analysis. **(A)** Kaplan-Meier survival analysis of the prognostic model signatures in the training set, testing set, and all TCGA dataset. **(B)** Kaplan-Meier survival analysis of the prognostic model signatures in the external validation sets (GSE13507, GSE31684, GSE32894, GSE69795). The survival curves demonstrate significant differences in survival probability between the high-risk and low-risk score groups across different external datasets, with p-values, hazard ratios, and 95% confidence intervals provided.

The model was first trained and tuned using the training set, and then its performance was evaluated internally and validated using the testing set. Additionally, the model was also validated using the entire dataset to further assess its performance and generalization ability. Within these assessments, the training set, testing set, and all datasets were categorized into high- and low-risk groups based on risk score. It was found that significant statistical differences in outcomes were evident in each set, with a notably worse prognosis in the training set (HR=3.4, P<0.001), the testing set (HR=2.45, P=0.001), and the all set (HR=3.11, P<0.001) among patients with high-risk score (**Figure 7A**). The model’s robustness was further demonstrated in four external validation sets, consistently yielding statistically significant results. Notably, unfavorable prognoses in GSE13507 (HR=1.99, P=0.003), GSE31684 (HR=1.66, P=0.039), GSE32894 (HR=7.26, P=0.002), GSE69895 (HR=inf, P=0.028) were exhibited by patients with high-risk score (**Figure 7B**).

**Figure 8:**
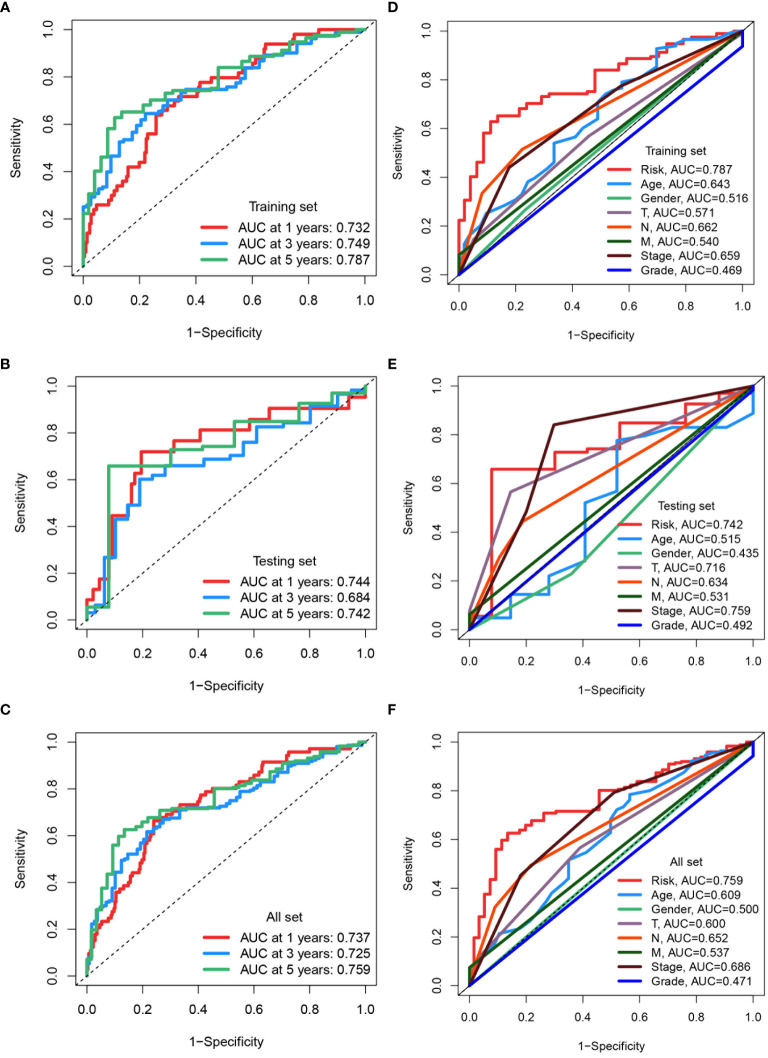
Assessing and comparing predictive accuracy of prognostic models. **(A-C)** ROC curves assessing the predictive accuracy of the prognostic model for 1-, 3-, and 5-year overall survival in the training set, testing set, and all TCGA datasets. The area under the curve (AUC) values are indicated for each time point. **(D-F)** ROC curves comparing the predictive capacity of the risk score to clinical factors in the training set, testing set, and all TCGA datasets. The AUC values for each factor are provided.

ROC curves were used to assess the predictive accuracy of the constructed prognostic model, yielding AUC values for 1-, 3-, and 5-year overall survivals in the training set: 0.732, 0.749, and 0.787 (**Figure 8A**). In the testing set, corresponding AUC values were 0.744, 0.684, and 0.742 (**Figure 8B**), while in the entire dataset, they were 0.737, 0.725, and 0.759, respectively (**Figure 8C**). To further validate the predictive capacity of the risk score in conjunction with other clinical features, comprehensive assessments were conducted. The risk score exhibited the highest predictive accuracy with an AUC of 0.787, outperforming other clinical factors such as age (AUC = 0.643), T stage (AUC = 0.571), N stage (AUC = 0.662), and overall stage (AUC = 0.759) in training Set (**Figure 8D**). Similar performances of risk score were also found in testing set (AUC = 0.742) (**Figure 8E**) and entire set (AUC = 0.759) (**Figure 8F**). The risk score demonstrated superior prognostic capability compared to factors such as age, gender, T stage, N stage, M stage and tumor grading was demonstrated by the risk score across all datasets.

**Figure 9:**
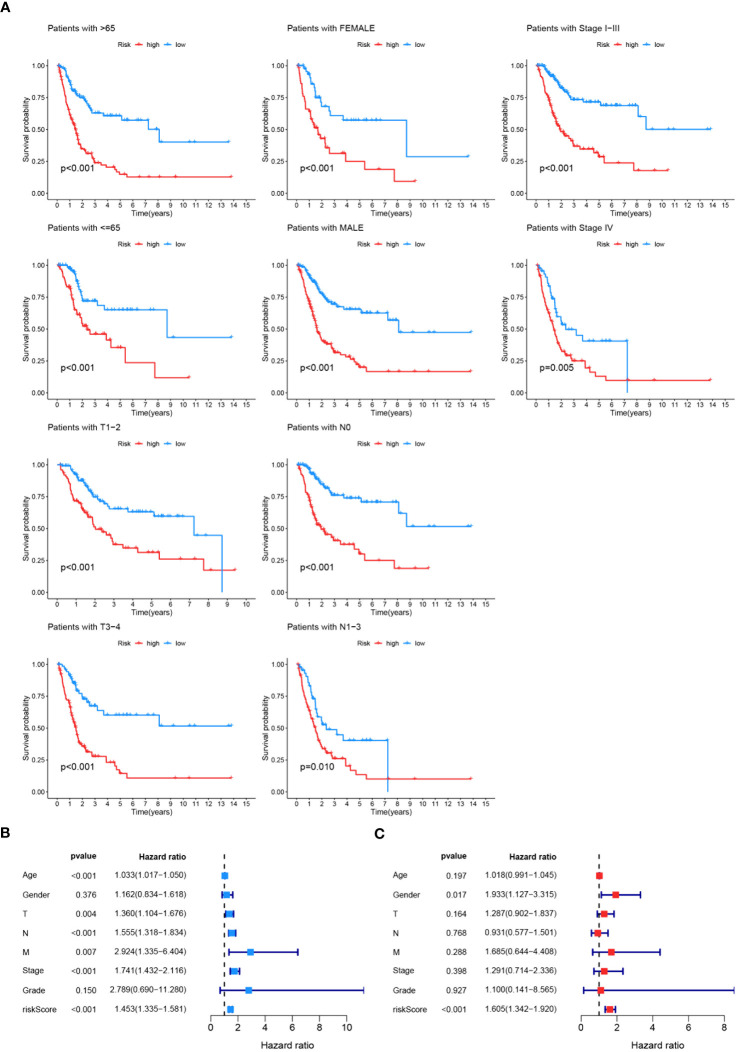
Validation and Evaluation of Prognostic Model Performance. **(A)** Kaplan-Meier survival analyses across various clinical features (age, gender, tumor stage, T stage, N stage, M stage) to validate the effectiveness of the prognostic model. The survival curves illustrate significant differences in survival probability between high-risk and low-risk groups within each clinical feature category, with p-values indicated for each subgroup. **(B)** Univariate Cox analysis assessing the prognostic significance of age, gender, T stage, N stage, M stage, tumor stage, grade, and risk score. **(C)** Multivariate Cox analysis evaluating the prognostic model as an independent indicator among different clinical signatures, with hazard ratios and p-values indicated.

**Figure 10:**
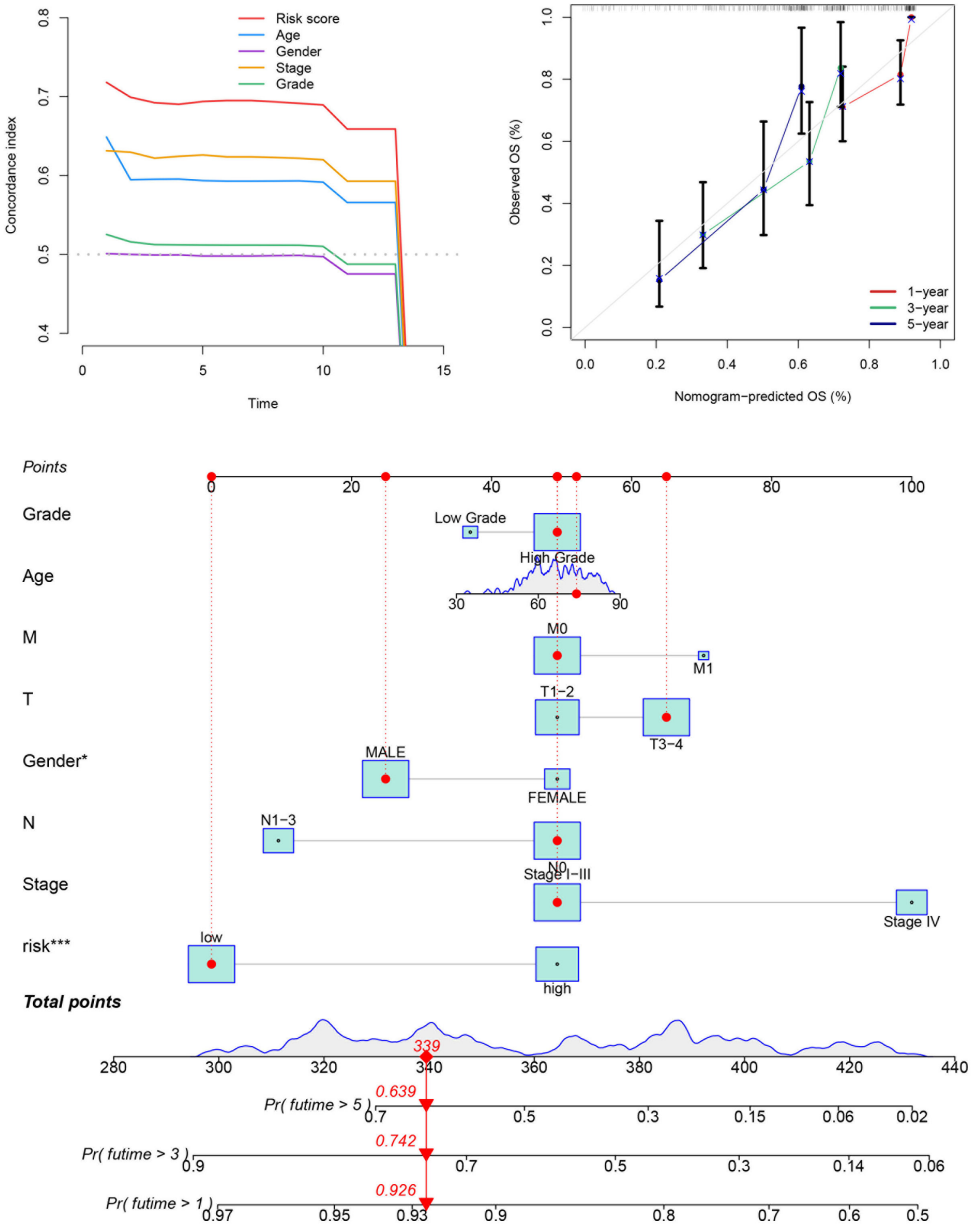
Evaluation and integration of the prognostic nomogram. **(A)** The plot displaying the prediction accuracy for both the risk score and various clinical signatures (age, gender, tumor stage, T stage, N stage, M stage, grade). The C-index over time is shown for each variable. **(B)** The calibration curve used to assess the predictive accuracy of the nomogram, comparing the predicted OS at 1-, 3-, and 5-year intervals with the observed OS. **(C)** A nomogram integrating the prognostic model with clinical variables such as grade, age, M stage, T stage, gender, N stage, clinical stage, and risk score. The nomogram provides a total points score to predict the probability of 1-, 3-, and 5-year overall survival. *P<0.05, ***P<0.001.

The effectiveness of the prognostic model across patients with diverse clinical features, including diverse age (>65 and ≤65), genders (female and male), pathological stages (I-III), T stages (1-2 and 3-4), and lymphatic metastasis statuses (N0 and N1-3) was validated. It was demonstrated that better survival outcomes were associated with patients with low-risk score (**Figure 9A**). The prognostic significance of age (p<0.01), T stage (p<0.01), N stage (p<0.01), M stage (p<0.01), tumor stage (p<0.01), and risk score (p<0.01) (**Figure 9B**) was confirmed by univariate Cox analyses. The risk score (p<0.01) was evaluated as an independent indicator among different clinical signatures by multivariate Cox analysis (**Figure 9C**). The prediction accuracy of the risk score and clinical signatures (age, gender, stage, grade) was assessed over time using the C-index. The risk score demonstrated better performance in predicting survival prognosis compared to other clinical factors. (**Figure 10A**). A nomogram that incorporates clinical characteristics (grade, age, M stage, T stage, gender, N stage, clinical stage) and risk score was created to predict 1-year, 3-year, and 5-year survival rates for BLCA patients (**Figure 10C**). Additionally, the calibration curve showed strong consistency between predicted survival and actual outcomes (**Figure 10B**).

### 3.4 Risk score stratification predicts immunotherapy response in TCGA-BLCA

**Figure 11:**
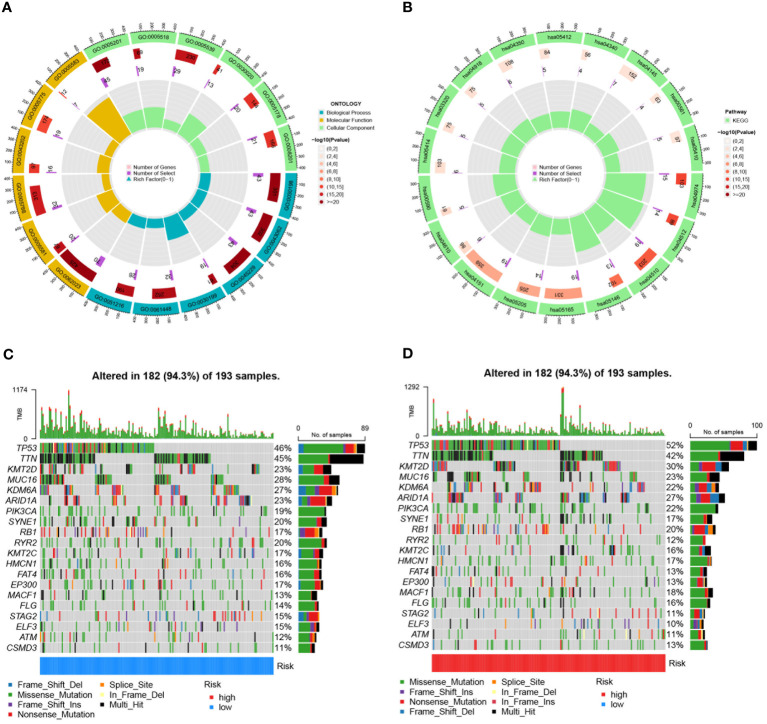
Enrichment analyses and mutation profiles in TCGA-BLCA based on risk score groups. **(A)** The GO enrichment results for TCGA-BLCA based on risk score groups. **(B)** KEGG pathway enrichment analysis for TCGA-BLCA based on risk score groups. **(C)** The top 20 mutated genes in the low-risk group of TCGA-BLCA. **(D)** The top 20 mutated genes in the high-risk group of TCGA-BLCA.

Differential expression analysis was conducted on TCGA-BLCA samples based on risk score, followed by GO and KEGG analyses on the differentially expressed genes. The main Biological Process terms identified in GO included GO:0030199: extracellular matrix structural constituent, GO:0043062: extracellular structure organization, GO:0045229: external encapsulating structure organization (**Figure 11A**). The KEGG pathways were primarily enriched in HSA04151: PI3K-Akt signaling pathway, hsa04512: ECM-receptor interaction, hsa04510: Focal adhesion (**Figure 11B**). Mutation frequencies were quantified in two groups, presenting the top 20 mutated genes. The top mutated genes in the low-risk group included TP53, TTN, KMT2D, MUC16, and KDM6A, with mutation rates of 46%, 45%, 23%, 23%, and 22%, respectively (**Figure 11C**). The top mutated genes in the high-risk group also included TP53, TTN, KMT2D, and MUC16, but with higher mutation rates of 52%, 42%, 30%, and 27%, respectively (**Figure 11D**).

**Figure 12:**
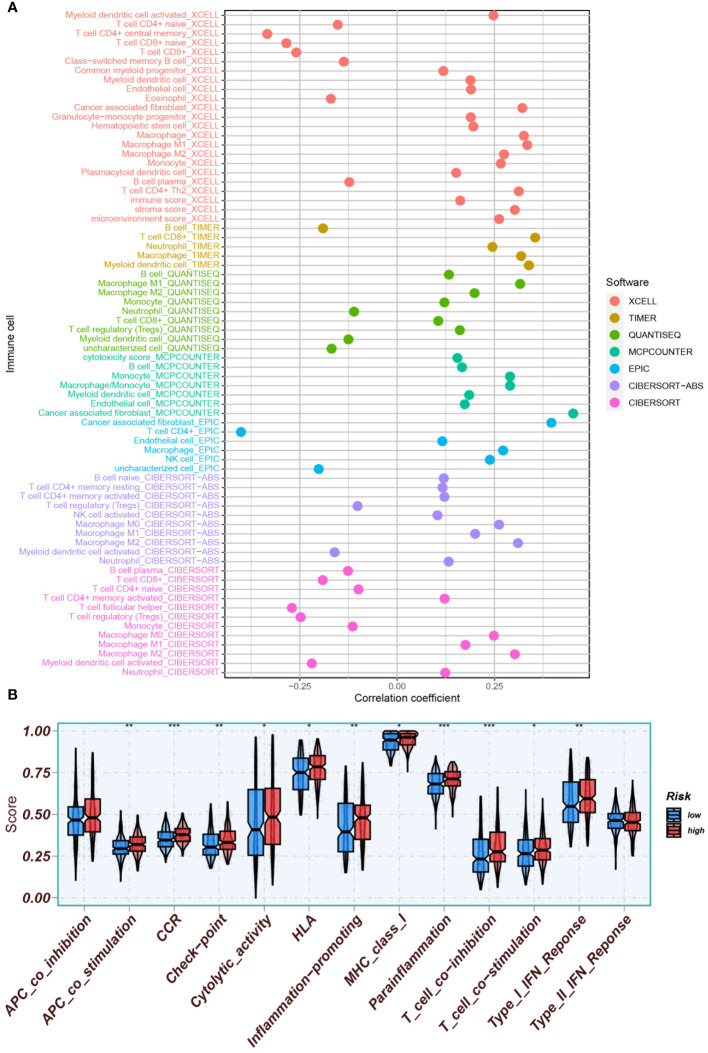
Assessment of the tumor immune microenvironment. **(A)** Immune cell infiltration was assessed using various software tools (XCELL, TIMER, QUANTISEQ, MCPCOUNTER, EPIC, CIBERSORT-ABS, CIBERSORT) to enhance evaluation accuracy. **(B)** ssGSEA was conducted to identify immune function differences between high- and low-risk groups. *P<0.05, **P<0.01, and ***P<0.001.

**Figure 13:**
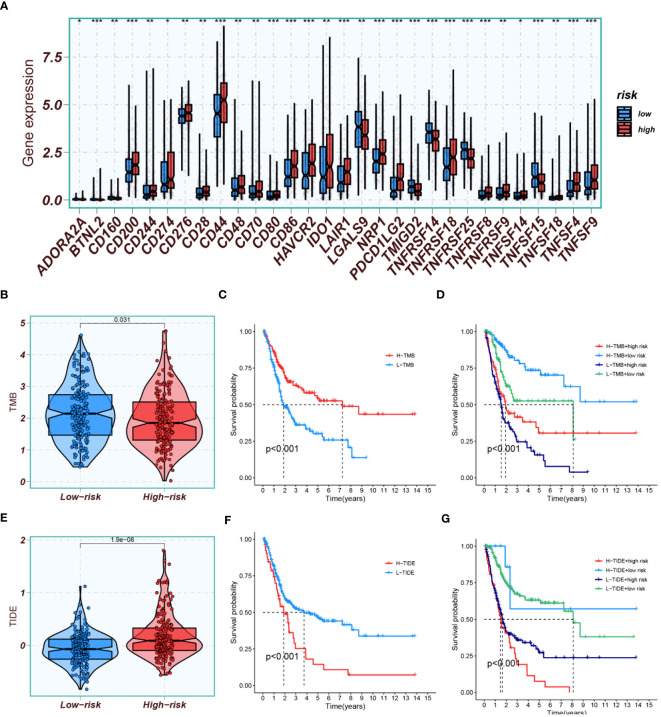
Immune profiling and prognostic analyses. **(A)** Comparison of immune checkpoint gene expression between high-risk and low-risk groups. **(B)** A plot displaying TMB scores in both high- and low-risk groups. **(C)** Kaplan-Meier analysis comparing prognosis between groups with high and low TMB scores. **(D)** Kaplan-Meier analysis evaluating survival based on combinations of high/low TMB scores with high/low risk groups. **(E)** A plot depicting TIDE scores in both high- and low-risk groups. **(F)** Kaplan-Meier analysis comparing prognosis between groups with high and low TIDE scores. **(G)** Kaplan-Meier analysis assessing survival based on combinations of high/low TIDE scores with high/low risk groups. *P<0.05, **P<0.01, and ***P<0.001.

Potential immunotherapy strategies were explored following this. Macrophages, myeloid dendritic cells, and neutrophils were found to be positively correlated with the risk score through Spearman correlation analysis (**Figure 12A**). Substantial differences in immune function were identified by ssGSEA, with especially pronounced variances observed in CCR, parainflammation, and T cell co-suppression, along with higher scores noted in the high-risk group for check-point, MHC-class-I, HLA, etc. (**Figure 12B**). Furthermore, the expression of immune checkpoint genes were compared between high-risk and low-risk groups. High-risk groups exhibited significantly higher expression of several immune checkpoint genes, including CD274, CD276, and CD44. Conversely, it was found that the expression levels of LGALS9, TNFRSF14, and TNFRSF25 were higher in the low-risk group compared to the high-risk group (**Figure 13A**).

TMB results indicated that the TMB scores of the low-risk group were generally higher than those of the high-risk group (**Figure 13B**). High TMB scores suggested a higher mutation burden in tumors, making the cancer cells more recognizable and attackable by the immune system. Kaplan-Meier analysis showed that the prognosis was better in the group with high TMB scores than in the group with low TMB scores (**Figure 13C**). The best survival prognosis was observed with a combination of high-TMB and low-risk, while the worst prognosis was associated with a combination of low-TMB and high-risk (**Figure 13D**).TIDE results demonstrated that the scores were lower in the low-risk group than in the high-risk group (**Figure 13E**), suggesting that patients in the low-risk group were more likely to be sensitive to immunotherapy. Survival analysis indicated that the survival prognosis with low-TIDE was better than with high-TIDE (**Figure 13F**). The best survival prognosis was associated with a combination of low-TIDE and low-risk, and the worst was with a combination of high-TIDE and high-risk (**Figure 13G**).

**Figure 14:**
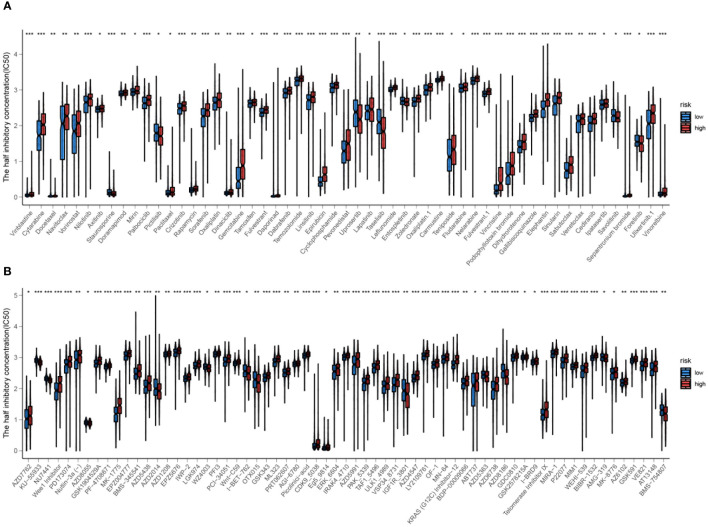
**(A, B)** Prediction of drug responsiveness in high and low-risk groups to various systemic drugs. The IC50 values for different drugs are compared between the high-risk and low-risk groups. Statistical significance is indicated by *P<0.05, **P<0.01, and ***P<0.001.

The analysis was extended to calculate the IC50 values of various systemic agents. The responsiveness to various systemic drugs was predicted and compared between high-risk and low-risk groups by analyzing the IC50 values. Interestingly, most drugs, like gemcitabine, oxaliplatin and rapamycin, showed increased efficacy in the low-risk group. Notably, inhibitors of PI3K and PI3Kβ, such as Tasilisib and Pictilisib, displayed superior effectiveness and sensitivity in the high-risk group, attributed to their notably lower IC50 values (**Figures 14A, B**).

### 3.5 Differential expression of prognostic genes

**Figure 15:**
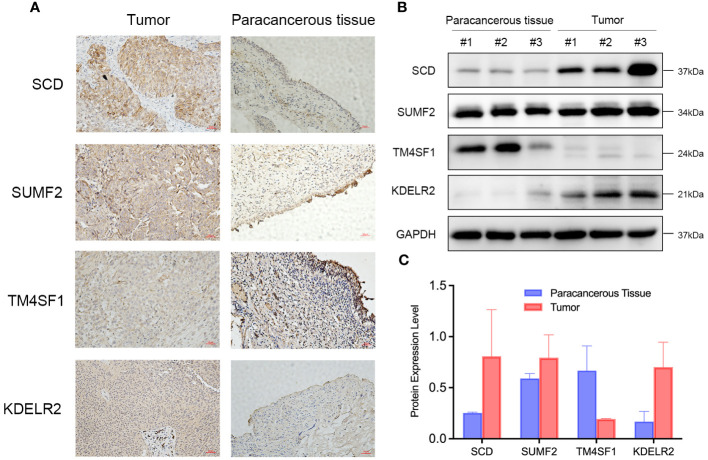
Differential Expression of SCD, SUMF2, TM4SF1, and KDEL2R in Bladder Cancer Tissues Compared to Paracancerous Tissues. **(A)** Immunohistochemical staining showed the expression of SCD, SUMF2, TM4SF1, and KDELR2 in the tumor and paracancerous tissue. **(B)** Western blot showed the expression of SCD, SUMF2, TM4SF1, and KDELR2 in three pairs of paracancerous tissues (#1, #2, #3) and corresponding tumor tissues (#1, #2, #3). **(C)** Quantification of protein expression levels from western blot results.

To investigate the expression of signature genes in the prognostic model, immunohistochemical staining and Western blot analysis on bladder cancer tissues and adjacent paracancerous tissues were performed. Immunohistochemical staining revealed that SCD, SUMF2, and KDEL2R were significantly more expressed in bladder cancer tissues compared to paracancerous tissues, while TM4SF1 exhibited higher expression in paracancerous tissues than in tumor tissues (**Figure 15A**). Western blot analysis further confirmed the differential expression of these proteins. Bladder cancer tissues from three different patients (#1, #2, and #3) exhibited markedly higher levels of SCD, SUMF2, and KDEL2R compared to the corresponding paracancerous tissues (**Figure 15B**). Conversely, TM4SF1 expression was higher in paracancerous tissues than in tumor tissues. GAPDH was used as a loading control to ensure equal protein loading across the samples. Quantitative analysis of the Western blot data visually confirmed the differential expression patterns observed, with higher levels of SCD, SUMF2, and KDEL2R in tumor tissues, and higher levels of TM4SF1 in paracancerous tissues (**Figure 15C**).

## 4 Discussion

BLCA, recognized as a common urological tumor, received significant attention for its treatment regimens (43). Although current adjuvant therapies effectively improved treatment outcomes for patients with BLCA, the pursuit of personalized treatment for each patient remains a primary focus for researchers (44). With the advancements in scRNA-seq and bulk-RNA seq, various subtypes of bladder tumors were identified over the years. However, the practical application of these subtypes in personalized treatments was not fully explored. Consequently, this study aimed to develop a prognostic model specifically for BLCA subtypes and to further investigated tailored treatment strategies informed by these subtypes.

Although various classifications of subtypes in BLCA were categorized by different researchers, they often involved basal-like and luminal types as foundational categories (45). The Ba/Sq subtype was more commonly observed in females and was frequently associated with higher stages of BLCA (stages pT3–4) (46). This subtype was characterized by heightened expression of basal and stem-like markers, including CD44, KRT5, KRT6A, KRT14, as well as squamous differentiation markers such as TGM1, DSC3, and PI340. These characteristics suggested similarities between the Ba/Sq subtype and basal-like breast cancers, as well as squamous cell carcinomas of the head, neck, and lungs (9). Kamoun et al. (47) established a classification for MIBC, where Ba/Sq accounts for the largest proportion, at 35%, with a median survival of 1.2 years. The subtype also exhibited a lower rate of complete pathological response in patients with locally advanced BLCA compared to other subtypes. The Ba/Sq subtype was associated with poorer overall survival and lower rates of complete pathological response in locally advanced BLCA patients compared with luminal and stroma-rich tumors (48). Moreover, the Ba/Sq subtype was characterized by a greater degree of clonal expansion than the luminal subtypes (49). Ba/Sq tumors displayed higher infiltration of B Cell Receptor and T Cell Receptor compared to Luminal papillary tumors. Moreover, increased TCR richness and diversity were significantly associated with improved survival in the Ba/Sq subtypes (49).

Six molecular subtypes of BLCA were identified; notably, the high score of the Ba/Sq subtype was found to be significantly associated with a poor prognosis. whereas the high score of the metabolism subtype was significantly associated with a good prognosis. The metabolism subtype reflected the coordinated expression of a set of genes that influence various aspects of tumor biology. These genes, while grouped under the “metabolism” label, may not directly relate to cellular metabolic processes. The result may related to the specific expression patterns of these genes, where different levels of expression could significantly impact the prognosis of bladder cancer patients. Some may impact tumor growth, invasion capabilities, and responses to therapy, thereby contributing to an improved prognosis (50). Some may affect the tumor microenvironment, including the infiltration and activity of immune cells. This influence could enhance the anti-tumor immune response and contribute to better clinical outcomes (51).

Patients classified within the high-risk score group were typically found to exhibit poorer prognoses, making it pertinent to explore potential differences in immunotherapy responses among various risk groups. It was observed that macrophages, myeloid dendritic cells, and neutrophils showed positive correlations with the risk score. The Ba/Sq subtype of BLCA was characterized by poor treatment responsiveness and heightened immune cell infiltration, which was further compounded by higher PD-L1 expression on both tumors and immune cells compared to luminal and stroma-rich subtypes. This expression profile, which included elevated levels of PD-1 protein, suggested a distinct immunological environment that could potentially influence the efficacy of targeted therapies (48, 52). Consequently, the tumor microenvironment and immune correlations of BLCA based on the risk score were further investigated. In the high-risk score group, elevated expression levels of immune functional genes such as CCR, parainflammation, and T cell co-inhibition, as well as mutations in CD274, CD276, and HAVCR2, were found. Although higher immunocompetence was observed in the high-risk group, it also implied elevated immunosuppression. However, further investigation into prognosis differences revealed that the low-risk score group exhibited a high TMB score, while the high-risk score group displayed a high TIDE score. A high TMB score was indicative of enhanced tumor recognition and elimination by the immune system, potentially leading to improved immunotherapy responsiveness (53). Conversely, a high TIDE score suggested resistance to immunotherapy, indicative of poorer treatment outcomes and prognosis (54). Thus, patients within the low-risk score group were potentially more responsive to immunotherapy.

Apart from immunotherapy, chemotherapy was shown to exhibit therapeutic effectiveness. The basal subtype, inherently aggressive, was observed to have high sensitivity to cisplatin-based combination chemotherapy (55). The benefit derived from neoadjuvant chemotherapy (NAC) appeared to be influenced by molecular subtypes, with basal-like tumors exhibiting improved survival rates following NAC (23). This observation suggested a potential therapeutic avenue for this subtype, as the greatest enhancement in overall survival was noted in basal-like tumors compared to surgery alone. Consequently, it has been recommended that patients with basal-like tumors be prioritized for NAC (56). Furthermore, increased expression of certain genes, such as ITIH5, was associated with enhanced sensitivity to chemotherapy in squamous cell carcinoma lines (57). In our analysis, gemcitabine and oxaliplatin were found to have lower IC50 values in the low-risk score group, suggesting that patients in this group could achieve better outcomes with gemcitabine and oxaliplatin treatment. This enhanced response could lead to more effective tumor reduction and potentially longer survival.

Although we conducted drug screening using only computer experimental methods, further validation by basic and clinical experiments was lacking. Firstly, computer simulations might not have fully mimicked the complex biological environment in the human body, so the IC50 values derived might have deviated from the actual situation. Secondly, IC50 values only reflected the effects of drugs on cells, whereas in actual treatment, factors such as drug absorption, distribution, metabolism, and excretion might also have impacted the therapeutic effect, and these factors usually could not have been fully considered in computer simulations. Therefore, IC50 values derived from computer experiments alone needed to be combined with clinical trial data and clinical observations to guide clinical treatment more accurately.

The model comprises ten genes, among which four genes exhibit a HR greater than 1: SCD, TM4SF1, and SUMF2. KDELR2, a key driver of non-small cell lung cancer invasion and metastasis, can be effectively targeted by inhibiting matrix metalloproteases to suppress invasion, presenting a potential treatment strategy for non-small cell lung cancer (58). Increased SCD activity, which led to the synthesis of more monounsaturated fatty acids, was associated with potentially promoting cancer cell growth and infiltration. Inhibition of SCD activity was shown to significantly impede the proliferation and invasion ability of BLCA cells (59). Although research on SUMF2 in cancer remains limited, it was found to bind to IL-13 and independently inhibit IL-13 secretion in bronchial smooth muscle cells (60). Interestingly, our experimental findings revealed elevated TM4SF1 expression in paracancerous tissues, which contrasts with previous reports associating higher TM4SF1 levels with cancerous tissues (61). This discrepancy could stem from differences in sample characteristics, tissue preparation methods, or analytical techniques utilized across studies. Future investigations could focus on validating these findings using larger and more diverse patient cohorts.

While this article yielded promising results, its limitation was identified in the need for further validation. Despite favorable outcomes were demonstrated by the prognostic model in both internal and external validation datasets, additional validation in independent cohorts or clinical trials was deemed essential to confirm its reliability and clinical utility. Moreover, the study primarily focused on molecular signatures and immune correlations of molecular subtypes, necessitating further experimental validation to assess the actual therapeutic efficacy of identified targets or immunotherapy strategies.

## 5 Conclusion

A new prognostic model based on the BSSRGs was identified and validated, demonstrating robust performance in predicting prognosis for BLCA patients. The risk score emphasized the potential of personalized therapy, guided by patient stratification and immune profiles, to enhance treatment efficacy.

## Data availability statement

The datasets presented in this study can be found in online repositories. The names of the repository/repositories and accession number(s) can be found in the article/**Supplementary Material**.

## Ethics statement

The studies involving humans were approved by Biomedical Research Ethics Committee of Affiliated Hospital of Zunyi Medical University. The studies were conducted in accordance with the local legislation and institutional requirements. Written informed consent for participation in this study was provided by the participants’ legal guardians/next of kin.

## Author contributions

XH: Writing – original draft, Data curation. GD: Writing – original draft, Validation. YY: Writing – original draft. PS: Writing – original draft, Resources. SC: Writing – original draft. CC: Writing – original draft. TH: Writing – original draft, Validation. YZ: Writing – original draft. YT: Writing – original draft. DT: Writing – original draft. NZ: Writing – review & editing, Funding acquisition.
