# Supplementary material for: Advancing bladder cancer management: development of a prognostic model and personalized therapy
Source: Front Immunol. 2024 Jul 22;15:1430792. doi: 10.3389/fimmu.2024.1430792 (PMC11298345; doi:10.3389/fimmu.2024.1430792)

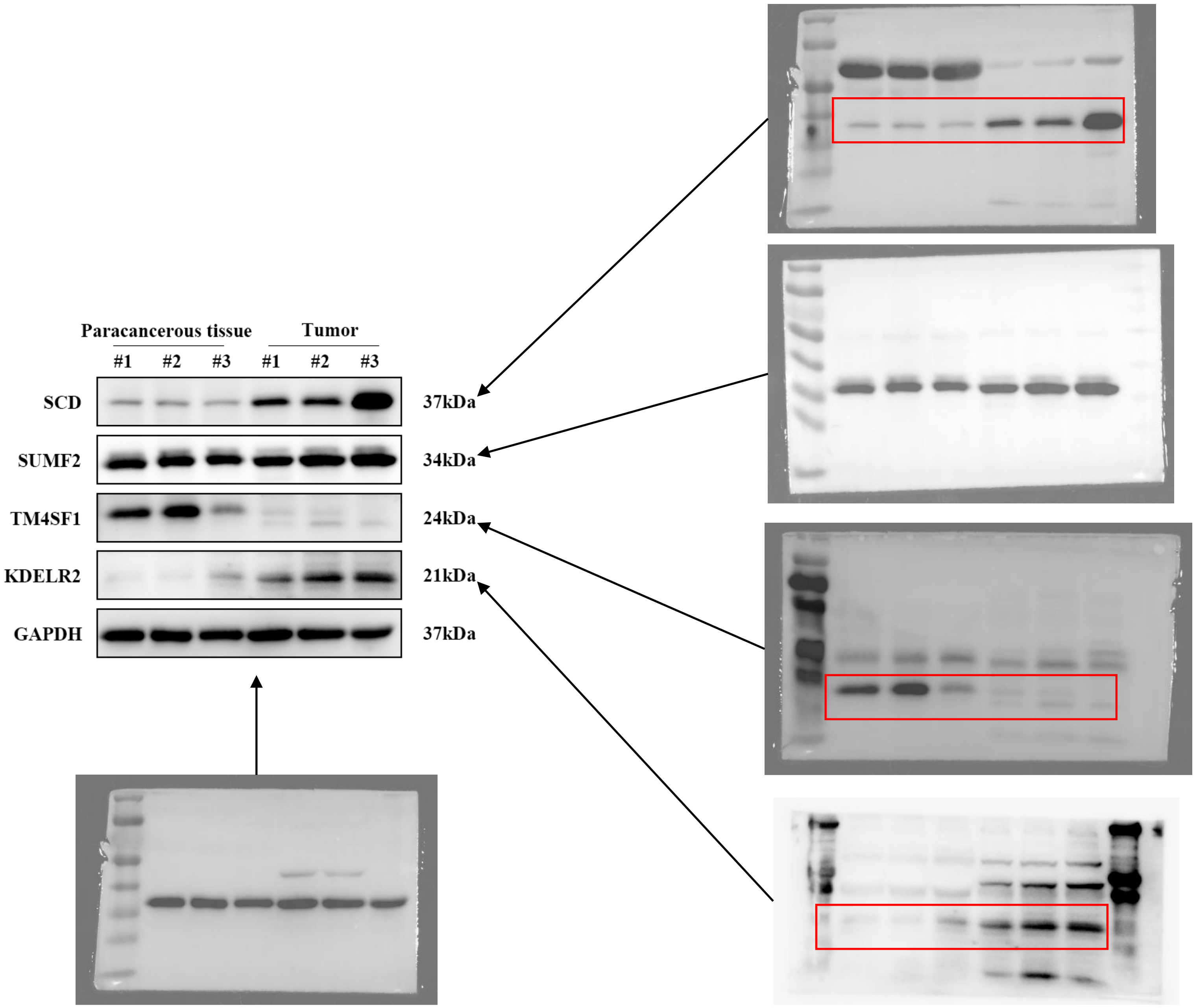

Paracancerous tissue
Tumor
#1
#2
#3
#1
#2
#3
SCD
37kDa
SUMF2
34kDa
TM4SF1
24kDa
KDELR2
21kDa
GAPDH
37kDa

SCD1(37kDa)

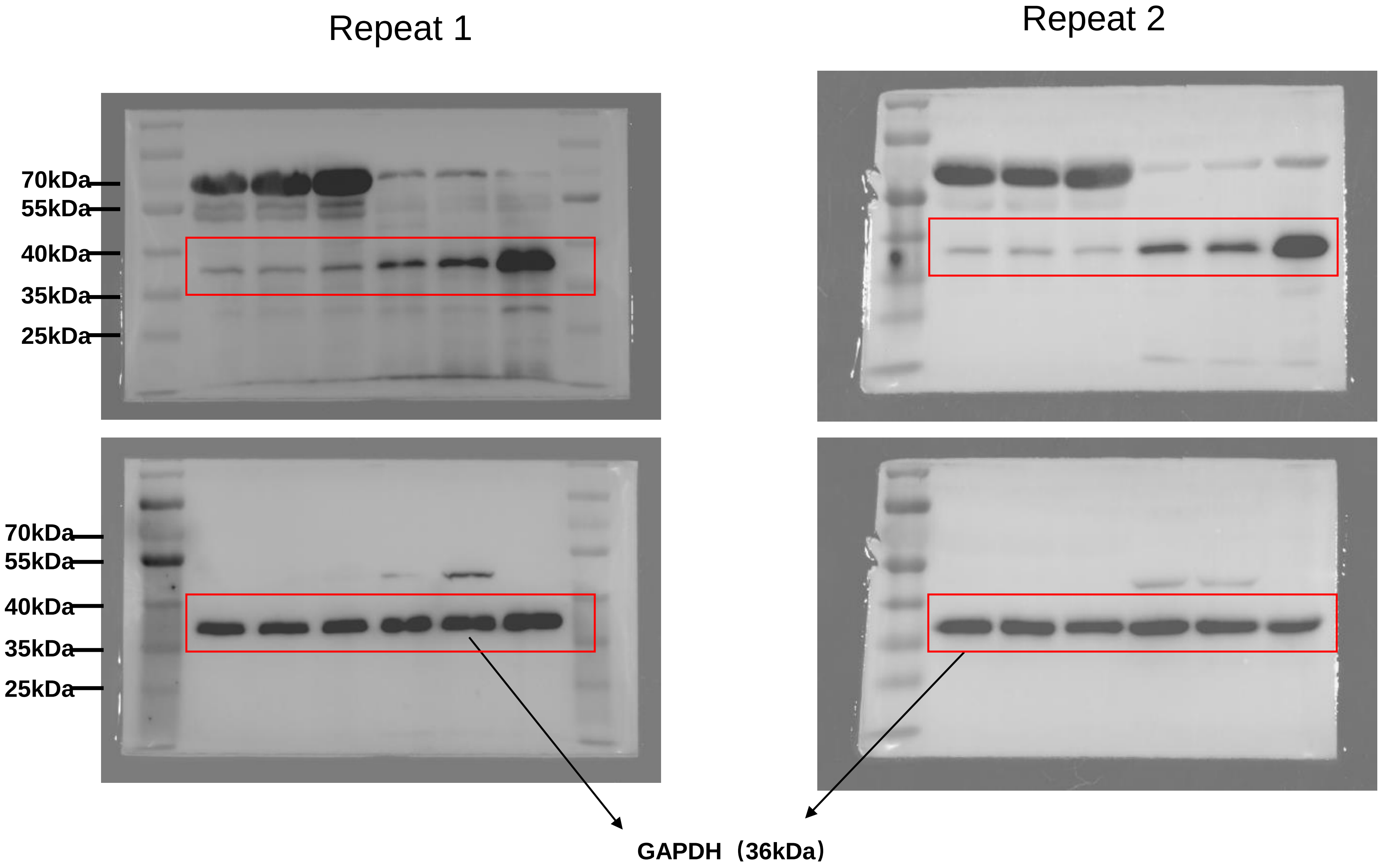
Repeat 1
Repeat 2
70kDa
55kDa
40kDa
35kDa
25kDa
70kDa
55kDa
40kDa
35kDa
25kDa
GAPDH（36kDa）

SCD1(37kDa)

Repeat 3

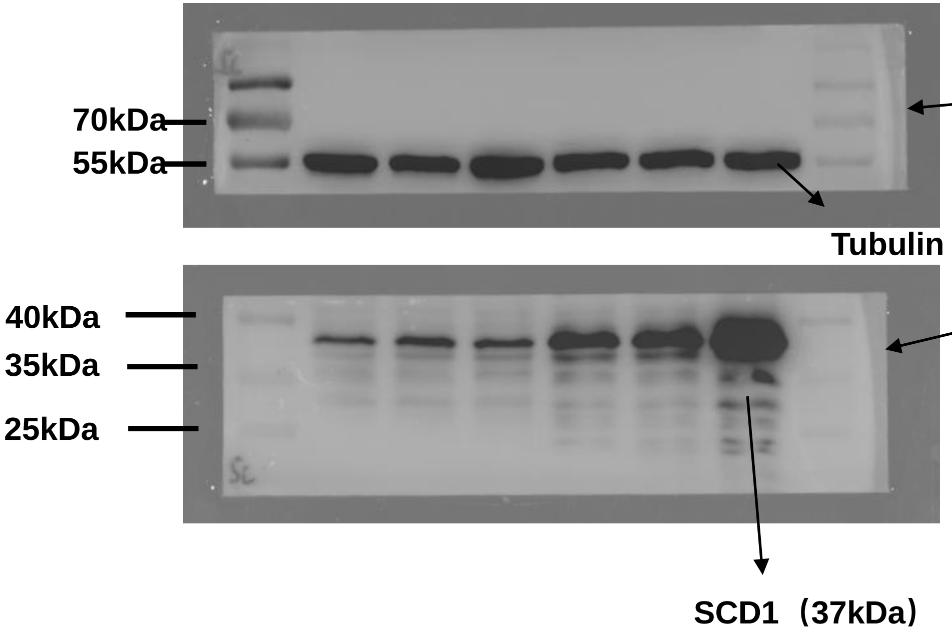

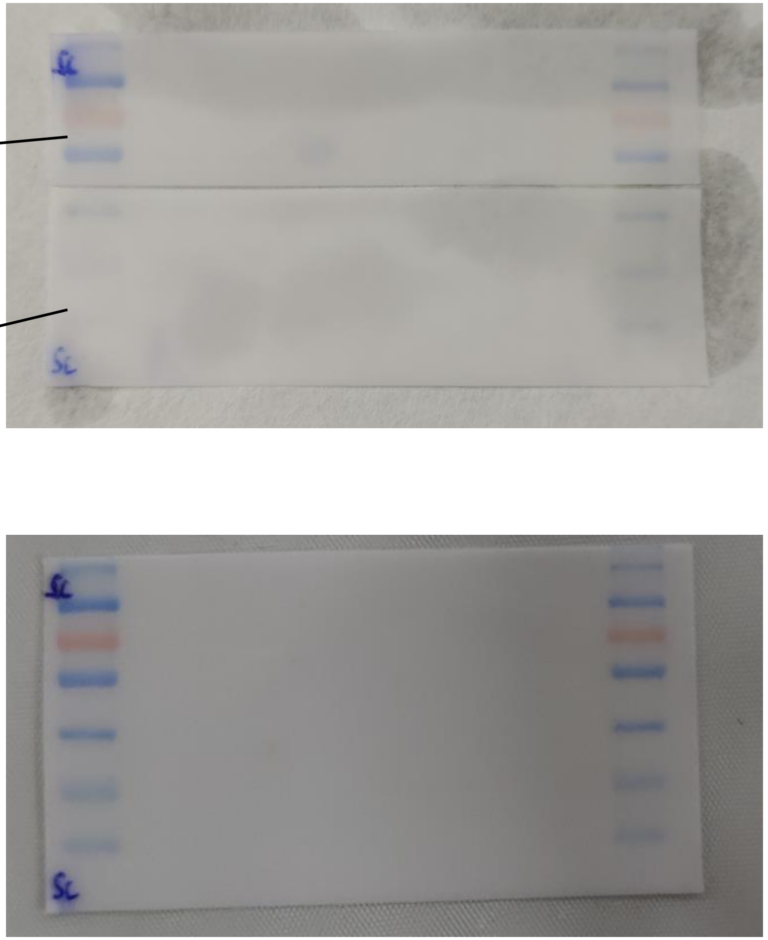

SUMF2(34kDa)

Repeat 1

Repeat 2

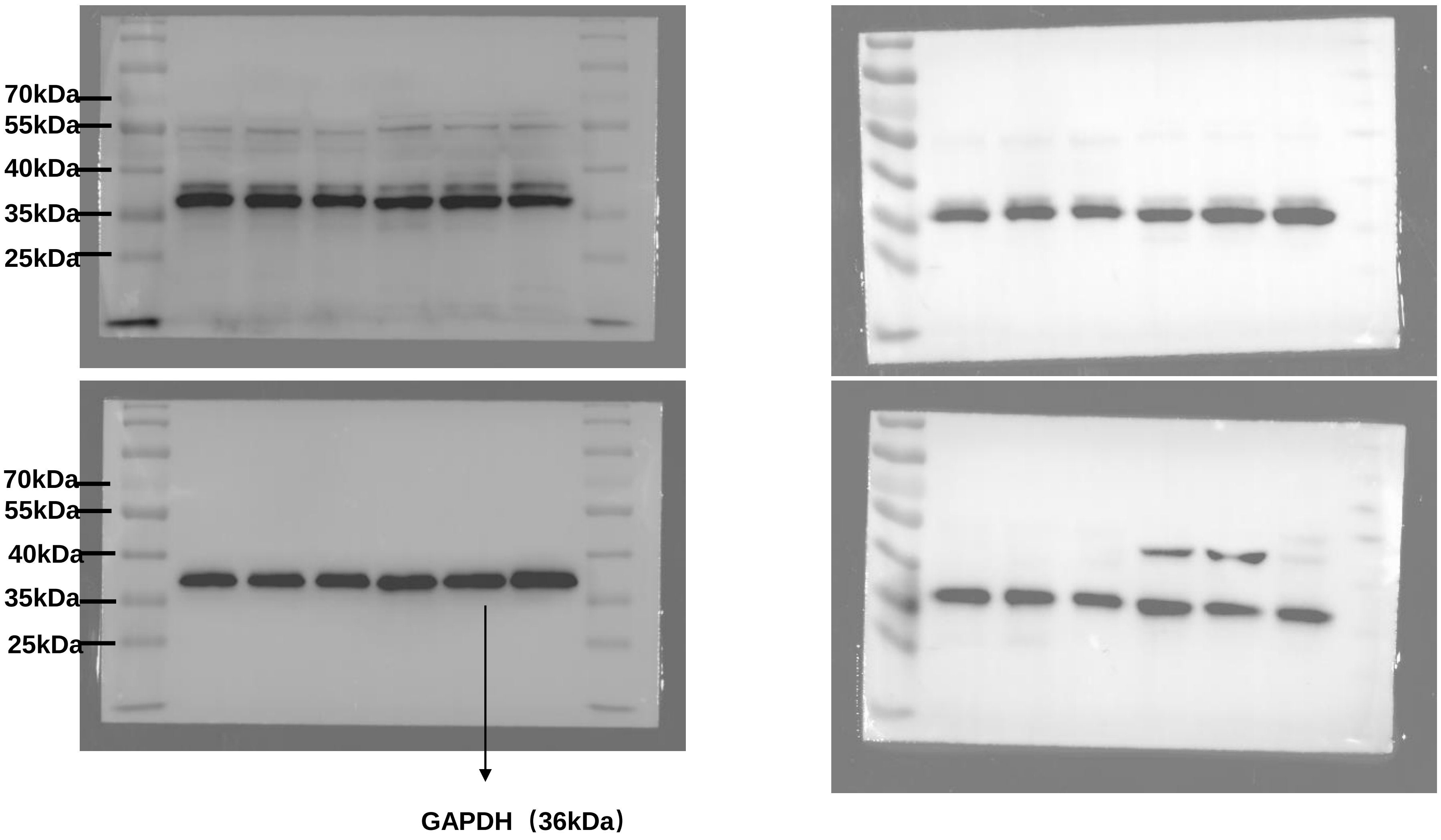

SUMF2(34kDa)

Repeat 3

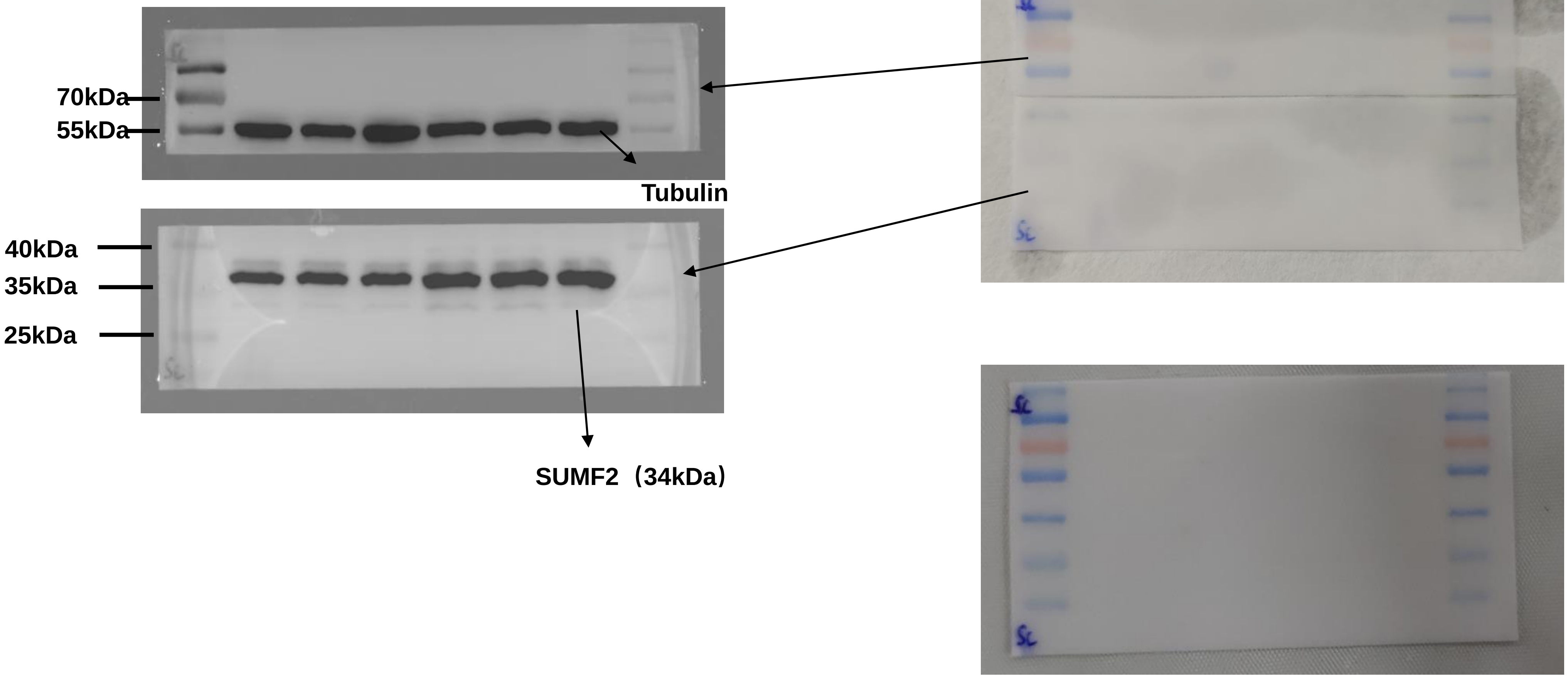

KDELR2(24kDa)， Observed:30kDa

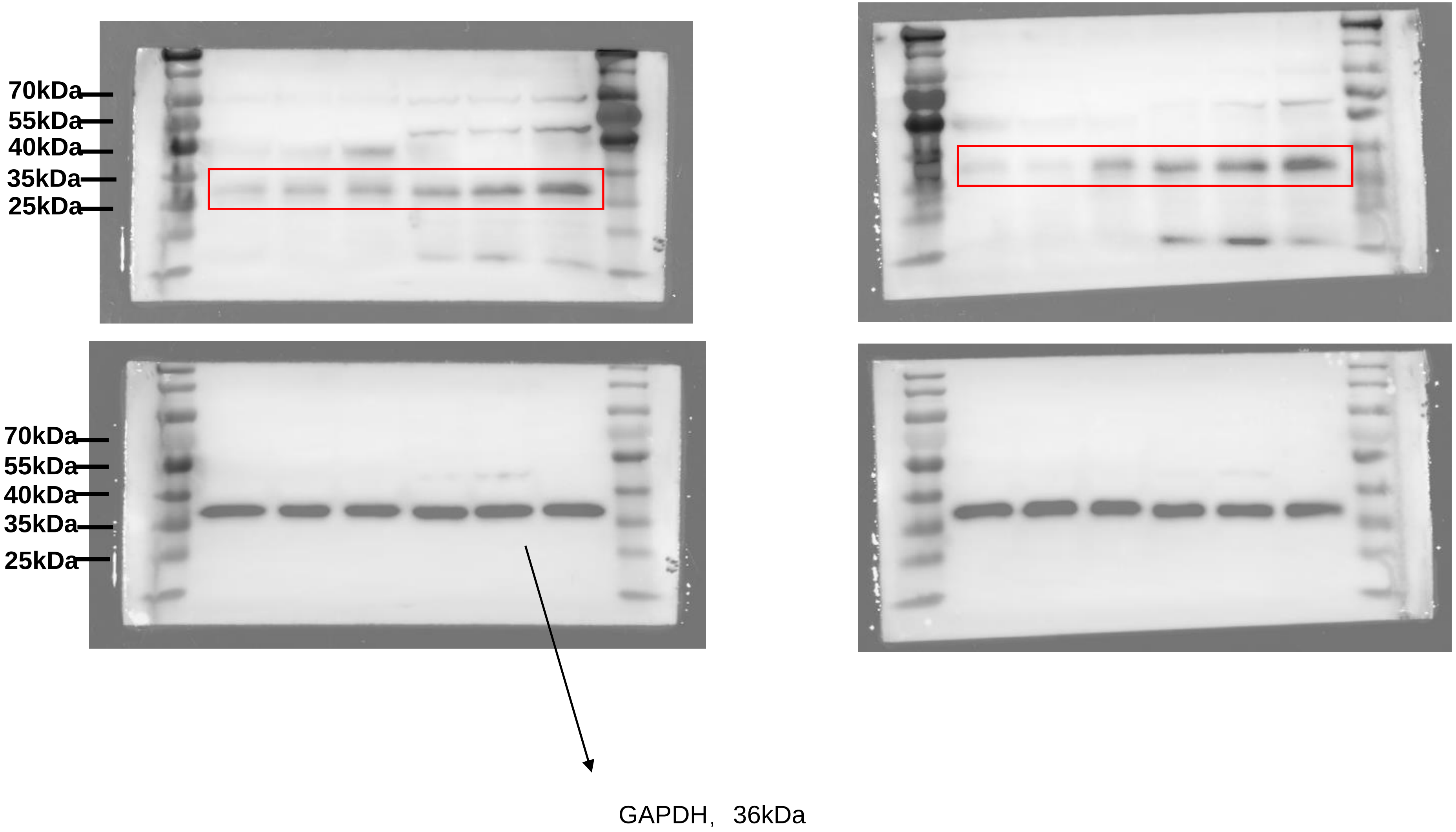

KDEL(24kDa)

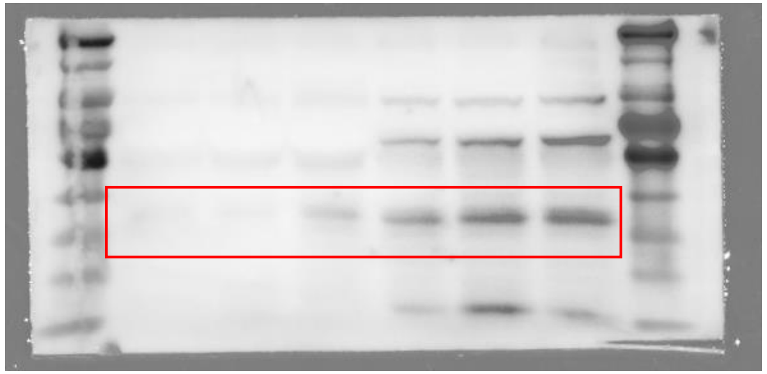

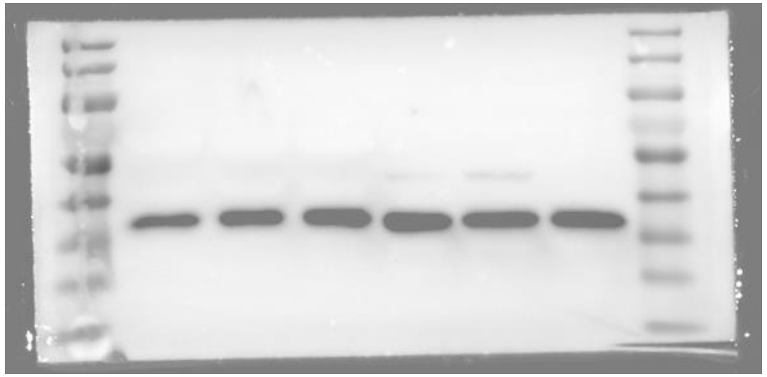

TM4SF1(21kDa)； Observed:28kDa

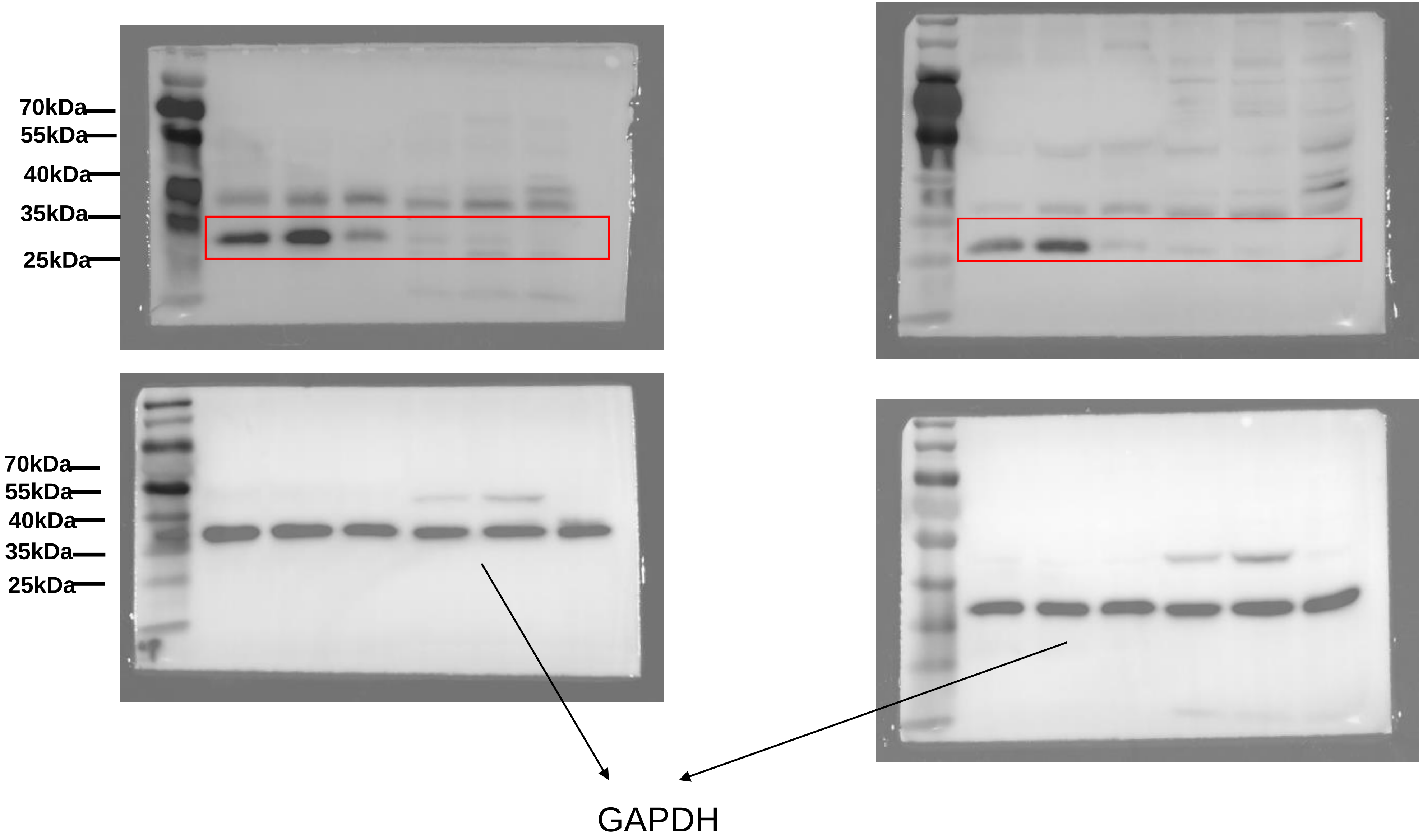

TM4SF1(21kDa); Observed:28kDa

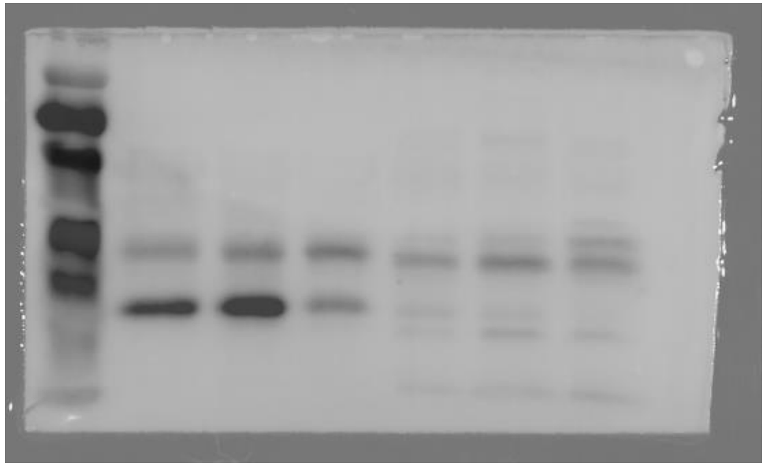

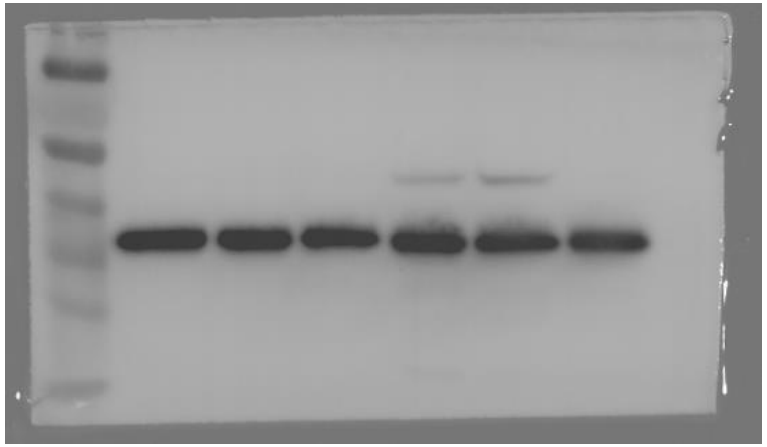

Supplement: Supplementary file 2 [file DataSheet_1.pdf]
